# INTRAVITREAL BEVACIZUMAB FOR THE TREATMENT OF CHOROIDAL HEMANGIOMAS

**DOI:** 10.1097/IAE.0000000000004323

**Published:** 2025-02-17

**Authors:** Hassan E. Elkayal, Mandeep S. Sagoo, Guy S. Negretti

**Affiliations:** * Ocular Oncology Service, Moorfields Eye Hospital, London, United Kingdom;; † Department of Ophthalmology, Faculty of Medicine, Alexandria University, Alexandria, Egypt; and; ‡ NIHR Biomedical Research Centre for Ophthalmology at Moorfields Eye Hospital and UCL Institute of Ophthalmology, London, United Kingdom.

**Keywords:** anti-VEGF, bevacizumab, circumscribed choroidal hemangioma

## Abstract

**Purpose:**

To investigate the use of intravitreal bevacizumab for the treatment of subretinal fluid (SRF) and intraretinal fluid associated with circumscribed choroidal hemangiomas.

**Methods:**

This was a retrospective review of all patients treated with at least three bevacizumab injections for circumscribed choroidal hemangiomas–associated SRF between May 2020 and August 2023 in Moorfields eye hospital. Outcome measures included change in best-corrected visual acuity, change in patient-reported symptoms, change in SRF, and change in central subfield thickness. Data on further management after cessation of injections were analyzed.

**Results:**

The study included nine patients. Median best-corrected visual acuity was 6/19 before and 6/24 after injections (*P* = 0.41). Central subfield thickness decreased from a median of 466 to 447 *µ*m (*P* = 0.11). Two thirds of (n = 6) patients did not show any reduction in foveal SRF, one third (n = 3) showed a partial reduction, and no patients had a complete resolution of SRF. Eight patients received rescue-photodynamic therapy, and one received external beam radiotherapy. Best-corrected visual acuity changed from a median of 6/60 to a median of 6/12 after rescue treatment (*P* = 0.63). The median central subfield thickness decreased significantly from 470 *µ*m to 249 *µ*m (*P* = 0.01).

**Conclusion:**

Intravitreal bevacizumab is unlikely to be an effective treatment for exudative circumscribed choroidal hemangiomas.

## Abstract 2

The use of intravitreal bevacizumab as an alternative to photodynamic therapy for the treatment of exudative choroidal hemangiomas was investigated. Bevacizumab treatment did not significantly improve the visual acuity, nor did it lead to the resolution of subretinal fluid. Bevacizumab is unlikely to be an effective treatment for choroidal hemangiomas.

Circumscribed choroidal hemangiomas (CCHs) are benign vascular hamartomas. Visual impairment can result if a CCH is associated with exudative changes in the macula, in the form of subretinal fluid (SRF) or intraretinal fluid (IRF). Circumscribed choroidal hemangiomas is likely congenital, but on average patients present with symptoms in the fifth or sixth decade of life.^1^ Photodynamic therapy (PDT) is currently considered the treatment of choice for visually significant exudative CCH,^1,2^ double duration PDT being more effective than single duration.^2^ Verteporfin (Visudyne, Cheplapharm Arzneimittel GmbH, Greifswald, Germany) is an intravenous dye that acts as a photosensitizer during PDT. After intravenous injection, verteporfin is internalized by endothelial cells in the retinal blood vessels. Photoactivation of the verteporfin by a nonthermal diode laser results in a photochemical reaction, releasing oxygen-free radicals that lead to occlusion of the targeted blood vessels in the CCH.^3^

Since May 2020, there has been an international shortage of Visudyne because of manufacturing issues.^4^ Between May 2020 and March 2022, none was available in the United Kingdom, and after this, supplies have been limited. This has greatly affected on the ability to treat patients with CCH. Alternative treatment options for CCH are available. These include external beam radiotherapy, plaque radiotherapy, and transpupillary thermotherapy. However, all these alternative options carry a higher risk of side effects and visual loss compared with PDT.^1^

Vascular endothelial growth factor (VEGF) is a proangiogenic cytokine that is known to play a role in the pathogenesis of many retinal vascular diseases. Its upregulation in certain retinal diseases contributes to increased vascular permeability, which leads to accumulation of SRF and IRF. Intravitreal injection of anti-VEGF agents has become a well-established, safe and effective treatment to reverse the increased vascular permeability in wet age-related macular degeneration, diabetic macular oedema, and retinal vein occlusion. Several case series have reported the use of intravitreal anti-VEGF, as bevacizumab and conbercept, as a primary or secondary treatment for exudative CCH.^5–8^ A study of cytokine levels in aqueous humor samples from patients with CCH has shown increased levels of VEGF.^9^ Given the low risk profile of these injections, the use of intravitreal injections of bevacizumab has been proposed as an alternative to PDT given the unavailability of Visudyne. This study aims to investigate the effectiveness of intravitreal bevacizumab as an alternative treatment for exudative CCH (off-label use).

## Materials and Methods

The medical records of the Ocular Oncology Service at Moorfields Eye Hospital were retrospectively reviewed for patients with a diagnosis of CCH between May 2020 and August 2023. This study was registered as a clinical audit (number 1187) at Moorfields Eye Hospital and as such did not require Institutional Review Board Approval. The study adhered to the tenets of the Declaration of Helsinki.

The diagnosis of CCH was established using slit-lamp biomicroscopy and multimodal imaging including ultra-widefield fundus photography, autofluorescence imaging, optical coherence tomography, fundus fluorescein angiography, indocyanine green angiography, and high frequency B-scan ultrasonography (US). All patients included in the study had visual symptoms related to the presence of exudative changes in the macula secondary to the CCH. All patients received at least three injections of intravitreal bevacizumab (1.25 mg/0.05 mL), 4 weeks apart and had at least one follow-up appointment 1 month after the last injection. The injections were performed with an aseptic technique in a dedicated intravitreal injection suite.

Demographic data collected included patient age, sex, ethnicity, and affected eye. Clinical and imaging data at baseline examination included best-corrected visual acuity (BCVA), history of previous treatment for the CCH, tumor location, largest basal diameter, tumor thickness on US, and tumor density on US. Optical coherence tomography features included presence of SRF over the tumor and in the fovea, presence of IRF in the fovea and central subfield thickness (CSFT). Intravitreal injection data included the number of bevacizumab injections received, the interval between injections, and any injection-related complication. Data on further management of the CCH after cessation of injections were also collected.

Outcome measures included BCVA 1 month after the last injection, change in patient reported symptoms, change in SRF over the tumor and the fovea, change in IRF, change in CSFT, and change in tumor thickness on US.

Statistical analysis was performed using the nonparametric Wilcoxon signed-rank test for paired data using the statistical software GraphPad Prism software version 10.0.3 (Jandel software, La Jolla, CA). When *P* values were <0.05 (two tailed), the differences were regarded as statistically significant (possibility of a Type I alpha error <5%).

## Results

Nine patients underwent intravitreal bevacizumab treatment between May 2020 and August 2023. Patient demographics and the baseline features of the CCHs are shown in Table 1. The mean age of patients undergoing treatment was 53 years (median 58, range 33–68). Presenting mean logarithm of the minimum angle of resolution (LogMAR) visual acuity was 0.6 (Snellen 6/24) (median 0.5 [Snellen 6/18], range 0.0–1.5 [Snellen 6/6–2/60]). Six (67%) of the patients had no treatment for the CCH before the intravitreal bevacizumab, while 3 (33%) patients were previously treated with PDT. The mean diameter of the CCHs was 6.8 mm as measured by ultra-widefield color imaging and 7 mm as measured by US. The mean thickness of the CCHs on US was 1.9 mm. Eight (89%) of the CCHs were in the macula. All the CCHs showed SRF in the fovea while 4 (44%) showed IRF in addition to the SRF.

**Table 1.** Intravitreal Bevacizumab for the Treatment of Choroidal Hemangiomas: Patient Demographics and Baseline Features

Demographics	Number (%), n = 9
Age (years) mean (median, [range])	53 (58, [33–68])
Sex	
Male	7 (78)
Female	2 (22)
Race	
White	4 (44)
Other	3 (33)
Not recorded	2 (22)
Eye	
Right	7 (78)
Left	2 (22%)
Baseline visual acuity	
Snellen mean (median, [range])	6/24 (6/18, [6/6–2/60])
LogMAR mean (median, [range])	0.6 (0.5, [0.0–1.5])
Previous treatment of the choroidal hemangioma	
None	6 (67%)
Photodynamic therapy	3 (33%)
Clinical and imaging features of choroidal hemangioma	
Diameter (ultra-widefield colour imaging), mm mean, (median, [range])	6.8 (7.0, [3.6–11.0])
Diameter (ultrasound), mm mean, (median, [range])	7.0 (7.4, [3.9–10.9])
Thickness (ultrasound), mm mean, (median, [range])	1.9 (2.0, [1.0–2.6])
Distance to foveola (from tumor edge), mm mean, (median, [range])	−0.7 (−1.3, [−3.5 to 2.2])
Distance to optic disc (from tumor edge), mm mean, (median, [range])	1.8 (0.9, [0–4.3])
Epicenter quadrant	
Macula	8 (89)
Superior	1 (11)
Temporal	0 (0)
Inferior	0 (0)
Nasal	0 (0)
Epicenter anteroposterior location	
Macula	8 (89)
Macula to equator	1 (11)
Equator to ora serrata	0 (0)
Internal reflectivity on ultrasonography	
High	2 (22)
Medium	6 (67)
Low	1 (11)
Lipofuscin on autofluorescence	
Yes	6 (67)
No	3 (33)
Fluid location on optical coherence tomography	
Subretinal	9 (100)
Intraretinal and subretinal	4 (44)

LogMAR, logarithm of the minimum angle of resolution.

**Fig. 1.**
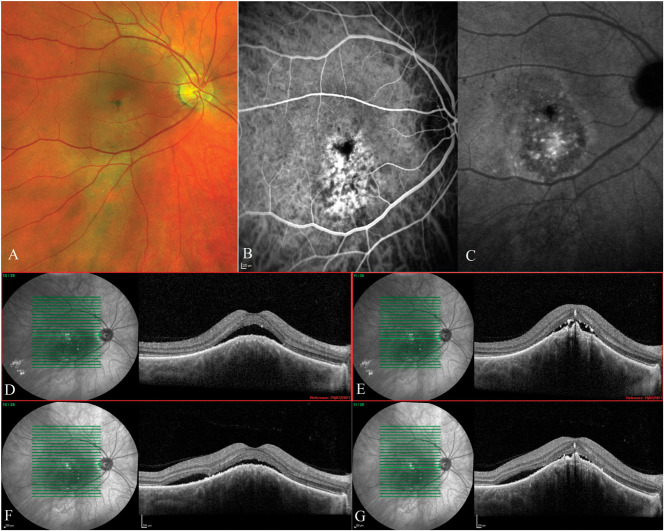
Male patient, 41 years old, presented with an exudative CCH. Best-corrected visual acuity was 6/9, and he reported distortion in vision. **A.** Widefield fundus image showing the CCH under the fovea. **B.** Early phase indocyanine green angiography image showing hypercyanescene of the CCH. **C.** Late phase indocyanine green angiography image showing partial washout of the dye from the CCH. **D** and **E.** Optical coherence tomography scans of the fovea at presentation showing SRF and hyperreflective subretinal deposits. **F** and **G.** Optical coherence tomography scans 1 month after three intravitreal injections of bevacizumab showing reduced height of SRF in the fovea and appearance of new SRF over the edge of the CCH. Best-corrected visual acuity remained at 6/9, and the patient reported partial improvement in his symptoms. The patient decided not to have any further treatment.

Outcomes after treatment of the CCHs with bevacizumab are shown in Table 2. Eight (89%) patients received three intravitreal injections of bevacizumab spaced 1 month apart, while 1 (11%) patient received a total of six injections. The median LogMAR BCVA was 0.5 (Snellen 6/18) (mean 0.6 [Snellen 6/24], range 0.0–1.5 [Snellen 6/6–2/60]) before injections and 0.6 (Snellen 6/24) (mean 0.6 [Snellen 6/24], range 0.0–1.8 [Snellen 6/6–1/60]) after injections (*P* = 0.41). The CSFT decreased from a median of 466 *µ*m (mean 478 *µ*m, range 377–587 *µ*m) to 447 *µ*m (mean 431 *µ*m, range 316–536 *µ*m) after injections (*P* = 0.11). Two thirds of (n = 6) patients did not show any reduction in foveal SRF, one third (n = 3) showed a partial reduction, and no patients had a complete resolution of SRF. Five patients had IRF at baseline, which did not improve after bevacizumab injections. The median thickness of the CCH was 2 mm (mean 1.8 mm, range 1.0–2.6 mm) preinjection, compared with 1.6 mm (mean 1.6 mm, range 0.8–2.7 mm) postinjections (*P* = 0.72). Seven (78%) patients reported persistent symptoms after treatment with injections, 1 (11%) patient reported worsening of their symptoms, while 1 (11%) patient reported improvement in their symptoms (Figure 1).

**Table 2.** Intravitreal Bevacizumab for the Treatment of Choroidal Hemangiomas: Outcomes

Treatment Outcomes of Intravitreal Bevacizumab	Number (%), n = 9
Follow-up (months) mean (median, [range])	15 (16, [8–23])
No. of injections	
3	8 (89)
6	1 (11)
Visual acuity (LogMAR)	
Preinjections mean, (median, [range])	0.6 (0.5, [0.0–1.5])
Postinjections mean, (median, [range])	0.6 (0.6, [0.0–1.8])
Wilcoxon signed-rank test	*P* = 0.41
OCT findings	
Central subfield thickness, *μ*m	
Preinjections mean, (median, [range])	478 (466, [377–587])
Postinjections mean, (median, [range])	431 (447, [316–536])
Wilcoxon signed-rank test	*P* = 0.11
Subretinal fluid in the fovea	
No change	6 (67)
Improved but not resolved	3 (33)
Subretinal fluid over the hemangioma	
No change	7 (78)
Worse	2 (22)
Cystoid macular oedema	
None present preinjections	4 (45)
No change	3 (33)
Worse	2 (22)
Ultrasound thickness, mm	
Preinjections mean, (median, [range])	1.8 (2.0, [1.0–2.6])
Postinjections mean, (median, [range])	1.6 (1.6, [0.8–2.7])
Wilcoxon signed-rank test	*P* = 0.72
Patient reported symptoms	
Persistent	7 (78)
Worse	1 (11)
Improved but not resolved	1 (11)

LogMAR, logarithm of the minimum angle of resolution; OCT, optical coherence tomography.

**Fig. 2.**
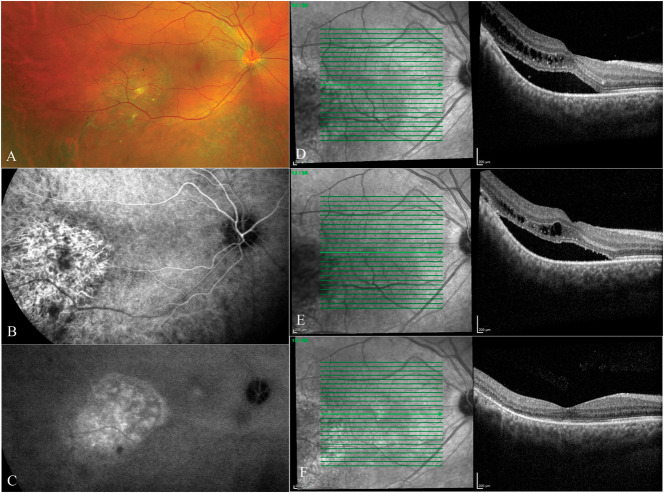
Male patient, 33 years old, presented with an exudative circumscribed CCH. Best-corrected visual acuity was 6/6, and he reported distortion in vision. **A.** Widefield fundus image showing the CCH in the inferotemporal part of the macula. **B.** Early phase indocyanine green angiography image showing hypercyanescene of the CCH. **C.** Late phase indocyanine green angiography image showing partial washout of the dye from the CCH. **D.** Optical coherence tomography scan of the fovea at presentation showing SRF and IRF. **E.** Optical coherence tomography scan 1 month after three intravitreal injections of bevacizumab showing persistent SRF and IRF. Best-corrected visual acuity remained 6/6. **F.** Optical coherence tomography scan after photodynamic therapy showing resolution of SRF and IRF. Best-corrected visual acuity improved to 6/5, and the patient's symptoms resolved.

The eight patients reporting persistent or worse symptoms had further treatment of the CCH, and their treatment outcomes are shown in Table 3. Seven patients received rescue treatment with double duration (166 seconds) PDT, while one patient received external beam radiotherapy. Of the patients receiving PDT, six patients required a single session of double duration PDT (Figure 2), while one patient required two sessions of double duration PDT spaced 5 months apart. The median interval between the first intravitreal injection and receiving rescue treatment was 6.5 months (mean 8.6 months, range 5–18 months). The LogMAR BCVA changed from a median of 1.0 (Snellen 6/60) (mean 0.8 [Snellen 6/36], range 0.2–1.8 [Snellen 6/9–1/60]) to a median of 0.3 (Snellen 6/12) (mean 0.8 [Snellen 6/36], range −0.1 to 2.1 [Snellen 6/5–Counting fingers]) (*P* = 0.63). The median CSFT decreased significantly from 470 *µ*m (mean 454 *µ*m, range 314–617 *µ*m) to 249 *µ*m (mean 263 *µ*m, range 171–419 *µ*m) (*P* = 0.01). The median thickness of the CCHs decreased significantly from 2 mm (mean 1.9 mm, range 1.0–2.6 mm) to 0.85 mm (mean 1.0 mm, range 0.3–2.2 mm) (*P* = 0.02). Two (25%) patients reported persistent symptoms, 5 (63%) patients reported improvement in their symptoms, and 1 (13%) patient reported resolution of their symptoms.

**Table 3.** Intravitreal Bevacizumab for the Treatment of Choroidal Hemangiomas: Postinjection Management of Choroidal Hemangiomas

Postinjection Management of Choroidal Hemangioma	Number (%), n = 8
Postinjection treatment modality	
PDT (1 session, double duration)	6 (75)
PDT (2 sessions, double duration)	1 (12.5)
EBRT	1 (12.5)
Visual acuity (LogMAR)	
Pre-further treatment mean, (median, [range])	0.8 (1.0, [0.2–1.8])
Post-further treatment mean, (median, [range])	0.8 (0.3, [−0.1 to 2.1])
Wilcoxon signed-rank test	*P* = 0.63
OCT findings	
Central subfield thickness, *μ*m	
Pre-further treatment mean, (median, [range])	454 (470, [314–617])
Post-further treatment mean, (median, [range])	263 (249, [171–419])
Wilcoxon signed-rank test	*P* = 0.01*
Subretinal fluid in the fovea	
Resolved	8 (100)
Subretinal fluid over the hemangioma	
Resolved	8 (100)
Cystoid macular oedema	
None present preinjections	3 (37.5)
Improved but not resolved	2 (25)
Resolved	3 (37.5)
Thickness of choroidal hemangioma, mm	
Baseline (US) mean, (median, [range])	1.9 (2.0, [1.0–2.6])
Post-further treatment (US or EDI-OCT) mean, (median, [range])	1.0 (0.85, [0.3–2.2])
Wilcoxon signed-rank test	*P* = 0.02*
Patient reported symptoms	
Persistent	2 (25)
Improved but not resolved	5 (62.5)
Resolved	1 (12.5)

* The result is significant at *P* < 0.05.

EDI, enhanced depth imaging; OCT, optical coherence tomography.

## Discussion

This study investigated the use of intravitreal injections of bevacizumab in the management of exudative CCH. There was no significant improvement in visual acuity (*P* = 0.41) nor a significant reduction of CSFT (*P* = 0.11) after treatment with bevacizumab. Two thirds of (n = 6) patients did not show any reduction in foveal SRF, one third (n = 3) showed a partial reduction, and no patients had a complete resolution of SRF. Most patients (n = 7, 78%) required rescue treatment with PDT when Visudyne could eventually be obtained. Despite a significant reduction of CSFT (*P* = 0.01) and complete resolution of foveal SRF in all patients after rescue PDT treatment, the visual acuity did not improve significantly (*P* = 0.63).

Several case reports and small series have suggested the beneficial effect of anti-VEGF in the treatment of exudative CCH. Sagong et al, reported on three cases of CCH treated with intravitreal bevacizumab. One case received a single injection of bevacizumab and showed resolution of SRF and visual improvement 1 month after the injection with no signs of recurrence during 8 months of follow-up. The other two cases received a single injection of bevacizumab as a pretreatment before PDT. Both cases showed an improvement in SRF 1 week after the injection, before the administration of PDT.^8^ Mandal et al, similarly reported on three cases. Two patients received two injections of bevacizumab 6 weeks apart, after nonresponse to transpupillary thermotherapy in one case and laser photocoagulation in the other. Both showed improvement in BCVA, serous detachment and cystoid macular oedema, which was maintained over 12 months of follow-up. The third patient received one injection of bevacizumab as a primary treatment for a CCH. Optical coherence tomography showed improvement in serous detachment after 6 weeks. The patient received a further injection in combination with laser photocoagulation. No further improvement was noted, and the initial response was maintained over 12 months.^7^

Subsequent studies have demonstrated that not all cases of CCH are responsive to anti-VEGF injections. A case report described a patient with an exudative CCH who showed no anatomical or visual improvement after two intravitreal injections of ranibizumab. The patient was subsequently treated with one session of PDT. The SRF resolved and the vision improved from 20/50 to 20/25.^10^ Kwon et al, reported on nine cases receiving intravitreal bevacizumab. Four patients received bevacizumab as a primary treatment for CCH, while five patients received it as a secondary treatment for recurrent or persistent SRF after transpupillary thermotherapy. Overall, 56% of the patients showed resolution of the SRF, while 44% had persistent SRF. There was a significant reduction of the median central foveal thickness from 514 *µ*m to 251 *µ*m. The median LogMAR visual acuity improved significantly from 0.7 to 0.5 after injections. Of the four patients receiving bevacizumab as a primary treatment, two showed resolution of the SRF while two showed persistent SRF. Of the five patients receiving bevacizumab as a secondary treatment, two showed resolution of the SRF, one showed persistent SRF and two showed worsening of the SRF. Intraretinal fluid was present in four out of these five patients. All four patients showed persistent IRF, despite two of them showing resolution of the SRF.^5^ Lai et al,^6^ prospectively investigated the effectiveness of intravitreal conbercept injections as a primary treatment for exudative CCH. Conbercept is an anti-VEGF recombinant fusion protein developed in China and approved by the Chinese Food and Drug Administration in 2013. It combines domains of the VEGF-receptor-1 and VEGF-receptor-2 to the Fc portion of human IgG-1.^11^ Lai et al, treated 42 patients, 55% of which were sensitive to conbercept showing resolution of the SRF. The mean number of injections was 3.83 in 6 months. The remaining 45% required rescue PDT or laser photocoagulation as they were nonresponsive after three injections of conbercept. At 3 months, the mean LogMAR visual acuity showed a nonsignificant improvement from 0.92 to 0.82, while the mean central foveal thickness decreased significantly from 534 *µ*m to 400 *µ*m for the whole group. Similar results were observed for the subgroup of patients responsive to conbercept. The mean LogMAR visual acuity showed a nonsignificant improvement from 0.84 to 0.76, and the mean central foveal thickness decreased significantly from 427 *µ*m to 259 *µ*m at the 6 months final follow-up. The same pattern was observed for the patients requiring rescue PDT. The mean LogMAR visual acuity showed a nonsignificant improvement from 1.08 to 0.71, and the mean central foveal thickness decreased significantly from 725 *µ*m to 418 *µ*m at 6 months.

The response to anti-VEGF injections was poor in our case series compared with most of the aforementioned studies. None of our patients showed resolution of SRF. The ethnic background of the patients included in these studies was different from ours. Kwon et al and Lai et al were treating patients of an East Asian background, while most of our patients were of a White background. Lai et al studied the effect of conbercept rather than bevacizumab; thus, the results are not directly comparable. Bevacizumab is a humanized monoclonal antibody that binds all isoforms of VEGF-A. Conbercept is an anti-VEGF recombinant fusion protein. It acts as a decoy receptor that binds to all isoforms if VEGF-A, VEGF-B, VEGF-C, and placental growth factor with higher affinity than bevacizumab.^11^

The thickness of the CCHs was not influenced by intravitreal bevacizumab injections but showed a significant reduction after rescue PDT. This is consistent with previous reports.^1,6^ The lack of a significant visual improvement after rescue PDT despite a good anatomical improvement might be related to the delay in availability of Visudyne. The delay between administering intravitreal bevacizumab and administering rescue PDT in our patients ranged from 5 months to 18 months. The chronicity of the exudative changes in the macula could have led to permanent damage of photoreceptors.

The current study has some limitations, specifically the small number of cases and its retrospective nature. In conclusion, contrary to previous studies, it appears that intravitreal bevacizumab is not an effective treatment option for exudative changes associated with a CCH. Based on the results of this study, enthusiasm for intravitreal bevacizumab as an alternative to PDT should be tempered, in favor of the latter for this indication. Larger studies are needed to verify these results and to explore the effectiveness of different anti-VEGF drugs.
